# Heart rate detection by Fitbit ChargeHR^™^: A validation study versus portable polysomnography

**DOI:** 10.1111/jsr.13346

**Published:** 2021-04-10

**Authors:** Davide Benedetti, Umberto Olcese, Paolo Frumento, Andrea Bazzani, Simone Bruno, Paola d’Ascanio, Michelangelo Maestri, Enrica Bonanni, Ugo Faraguna

**Affiliations:** ^1^ Department of Translational Research and of New Surgical and Medical Technologies University of Pisa Pisa Italy; ^2^ Center for Neuroscience Swammerdam Institute for Life Sciences University of Amsterdam Amsterdam the Netherlands; ^3^ Department of Political Sciences University of Pisa Pisa Italy; ^4^ Institute of Management Scuola Superiore Sant’Anna Pisa Italy; ^5^ Department of Clinical and Experimental Medicine University of Pisa Pisa Italy; ^6^ Department of Developmental Neuroscience IRCCS Fondazione Stella Maris Pisa Italy

**Keywords:** electrocardiography, photoplethysmography, wearables

## Abstract

Consumer “Smartbands” can collect physiological parameters, such as heart rate (HR), continuously across the sleep–wake cycle. Nevertheless, the quality of HR data detected by such devices and their place in the research and clinical field is debatable, as they are rarely rigorously validated. The objective of the present study was to investigate the reliability of pulse photoplethysmographic detection by the Fitbit ChargeHR^™^ (FBCHR, Fitbit Inc.) in a natural setting of continuous recording across vigilance states. To fulfil this aim, concurrent portable polysomnographic (pPSG) and the Fitbit’s photoplethysmographic data were collected from a group of 25 healthy young adults, for ≥12 hr. The pPSG‐derived HR was automatically computed and visually verified for each 1‐min epoch, while the FBCHR HR measurements were downloaded from the application programming interface provided by the manufacturer. The FBCHR was generally accurate in estimating the HR, with a mean (*SD*) difference of −0.66 (0.04) beats/min (bpm) versus the pPSG‐derived HR reference, and an overall Pearson’s correlation coefficient (*r*) of 0.93 (average per participant *r* = 0.85 ± 0.11), regardless of vigilance state. The correlation coefficients were larger during all sleep phases (rapid eye movement, *r* = 0.9662; N1, *r* = 0.9918; N2, *r* = 0.9793; N3, *r* = 0.9849) than in wakefulness (*r* = 0.8432). Moreover, the correlation coefficient was lower for HRs of >100 bpm (*r* = 0.374) than for HRs of <100 bpm (*r* = 0.84). Consistently, Bland–Altman analysis supports the overall higher accuracy in the detection of HR during sleep. The relatively high accuracy of FBCHR pulse rate detection during sleep makes this device suitable for sleep‐related research applications in healthy participants, under free‐living conditions.

## 1 INTRODUCTION

The commercial share of wrist‐worn “Smartbands” has grown rapidly in recent years. Such devices can collect physiological data in a user‐friendly and minimally invasive way, making them suitable for daily activity tracking (Henriksen et al., 2018). Smartbands have come to the medical community’s attention as tools capable of collecting biomarkers information, and therefore to monitor patients’ health status. Their role has been investigated in oncology, where they were tested as a tool for the activity tracking in breast cancer survivors (Chung et al., 2019), and in emergency departments, where they were tested for the low‐cost heart rate (HR) monitoring in critical patients (Dagan & Mechanic, 2020). Both studies concluded that commercial Smartbands are a feasible tool for HR monitoring in these two different circumstances. The possibility to collect data in a non‐invasive way accounts for the large spread of commercial Smartbands in sleep research and explains why there is a large amount of validation studies that address their accuracy (Henriksen et al., 2018). For instance, they have been used in population‐based projects to investigate circadian rhythms and sleep (Brazendale et al., 2019; Dunker Svendsen et al., 2019; Guarnieri et al., 2020; Lee & Finkelstein, 2015) and in autonomic nervous system (Dobbs et al., 2019; Hernando et al., 2018). Fitbit Inc. Smartbands are frequently used in research projects and are among the most popular wrist‐worn devices (Lewis et al., 2020). The FBCHR is one of the Fitbits Inc. Smartbands. It is equipped with a photoplethysmography sensor, a detector of microvascular oscillations of blood volume used to compute the HR. The FBCHR measurements have been validated in a plethora of different studies, as compared to electrocardiography (ECG) (Allen, 2007; Benedetto et al., 2018), polysomnography (PSG) (de Zambotti et al., 2016) and chest‐worn HR devices (Bai et al., 2018; Reddy et al., 2018). The accuracy of HR estimation was studied in different experimental settings, including both sedentary and physical activity conditions (Benedetto et al., 2018; Nelson & Allen, 2019) or during daily activity (Nelson & Allen, 2019). The FBCHR received the attention of sleep researchers interested in validating its HR measurements during sleep. Among them, de Zambotti et al., (2016) investigated the quality of HR detection of the FBCHR in sleep and compared its performance to the PSG‐based HR estimation. However, this experimental setting provides information on how FBCHR behaves under mostly sedentary conditions, as the data were collected in a controlled environment. Moreover, that study lacked sleep‐stage specificity as it did not consider the heterogeneity of HR across different sleep stages. In particular, the HR variability between vigilance stages could be considerably high, as well assessed by different studies (Penzel et al., 2003; Zemaityte et al., 1986). In fact, the mean HR tends to be lower in N1, N2, N3 stages compared to wakefulness, while in rapid eye movement (REM) stage the HR tends to be higher compared to N1, N2 and N3 stages. Haghayegh et al., (2019) addressed the quality of HR detection in each sleep phase, comparing the quality of HR measurement to the ECG with a 5‐min resolution. However, a temporal resolution of 5 min could not properly describe the sleep‐related HR dynamics (Penzel et al., 2003; Zemaityte et al., 1986).

In the present study, we aimed to address the quality of HR detection of the FBCHR in a natural setting, across all vigilance stages, with a 1‐min resolution, by comparing the HR data obtained from the manufacturer server with the portable PSG (pPSG)‐derived HR.

## 2 METHODS

### 2.1 Participants

A total of 25 volunteers were recruited among undergraduate and graduate students at the University of Pisa. The group comprised 17 females and eight males and the mean (*SD*) age was 22.36 (3.00) years. This study was approved by the University of Pisa Bioethical Committee (Review No: 02/2020 Prot. 0036352/2020).

### 2.2 Procedure

Participants were concurrently monitored with the FBCHR and a pPSG device. Once participants were equipped with the two devices, they were asked to carry on their daily routines without any specific activity restrictions. Participants spent ≥12‐hr wrist‐wearing under continuous (Nelson & Allen, 2019) monitoring.

### 2.3 Portable PSG

The pPSG recordings were performed through a portable device (MORPHEUS HOME LTM, Micromed). The electrodes were placed according to the American Academy of Sleep Medicine (AAMS) guidelines (Berry et al., 2012). We applied on the scalp 12 electroencephalographic (EEG) derivations electrodes (F3, F4, C3, C4, T3, T4, P3, P4, T5, T6, O1, O2, P, ground in Cz, reference in Fz), one ECG derivation on the chest, placed symmetrically around the sternum within the third and fourth ribs, two electrooculographic (EOG) (left and right vertical), and two electromyographic (EMG) derivations (electrodes placed on the chin over the suprahyoid muscles). Each 30‐s epoch was scored by a trained professional according to the AASM guidelines (Berry et al., 2012). The pPSG ECG measurements (sampled at 512 Hz) were imported and processed through a custom function in MATLAB (MATLAB R2020b, Math Works). The ECG signals were filtered through a Savitzky–Golay filter and a high‐pass filter. The Savitzky–Golay polynomial order (7) and frame length (30) were respectively maximised and minimised in order to improve the QRS complex quality (Hargittai, 2005). Due to a low‐frequency component in all 25 PSG’s ECG signals, we applied a high‐pass filter with a passband of 2 Hz.

The pPSG‐derived HR was then automatically computed by identifying the R peaks in each 1‐min epoch. The results of the computation were visually verified for 1‐min each epoch.

### 2.4 The FBCHR

A FBCHR Smartband is a wrist‐worn commercial device able to track the activity through a tri‐axial accelerometer sensor (micro‐electro‐mechanical systems [MEMS] tri‐axial accelerometer), whose measurements are used to compute steps and energy expenditure through a proprietary algorithm, and a photoplethysmography. The latter sensor can detect blood volume oscillations in the microvascular bed of a tissue, continuously. The collected data are shaped as a waveform, which is composed of a pulsatile component and a group of slow frequency components attributed to respiration, sympathetic nervous system activity, and thermoregulation. The former pulsatile component is generated by the cardiac synchronous changes in the blood volume with each heartbeat. The synchronicity of the pulsatile waveform to the blood volume is then used to compute the HR (Allen, 2007).

The FBCHR is equipped with a PurePulse^®^ light‐emitting diode (LED), a photoplethysmographic technology that could be found in other Fitbit Inc. devices, such as Fitbit Charge 2™, Fitbit Charge 3™, Fitbit Alta HR™, Fitbit Versa™, Fitbit Blaze™, and Fitbit Ionic™ (Haghayegh et al., 2019). We accessed FBCHR MEMS tri‐axial accelerometer and PurePulse^®^ measurements from the Fitbit Inc. server via the application programming interface (API), provided by Fitbit Inc. through a third‐party platform (www.sleepacta.com). Calorimetric and steps measures were stored at one data point per minute. As far as the HR data, Fitbit Inc. discloses that “Heart rate data is stored at one‐second intervals when in exercise mode and at five‐second intervals at all other times”, but does not officially disclose the HR sampling rate. However, the manufacturer does claim that the HR detectable range spans from 30 to 220 beats per min (bpm); a validation study could confirm that data are collected up to 153 bpm (Nelson & Allen, 2019), consistently with our data. The pPSG and FBCHR measurements were synchronised on the same computer when the participants were equipped with the devices, at the beginning of the recording session. Once data were imported in the MATLAB environment, they were graphically cross correlated to pPSG measurements for alignment (Buck et al., 2002).

### 2.5 Statistical analysis

Statistical analysis was performed in Python using NumPy library (Harris et al., 2020), while plots were computed through the Seaborn library.

**FIGURE 1:**
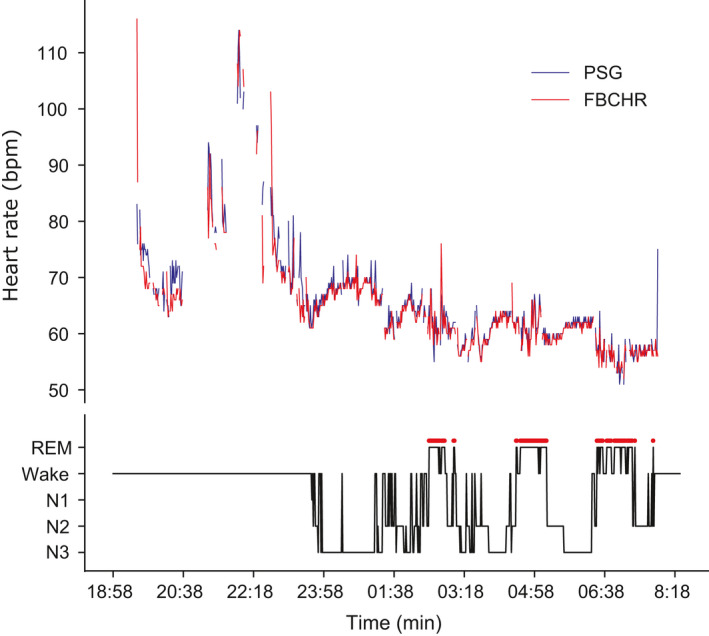
Minute‐by‐minute heart rate (HR) across the recording for a representative participant. Top panel: HR (beats/min [bpm]) derived from the portable polysomnographic (pPSG) electrocardiography (ECG) derivation (ECG, in blue) is plotted against the Fitbit ChargeHR™ (FBCHR, in red). Bottom panel: corresponding hypnogram, depicting the transitions across pPSG‐determined behavioural states (Wake, N1, N2, N3, rapid eye movement [REM])

While the FBCHR HR data were sampled at a 1‐min resolution, the pPSG were scored according to the AAMS criteria with a 30‐s resolution. We re‐coded pPSG epochs’ sleep score from 30‐s resolution to 1‐min resolution so that they were coupled (Sadeh et al., 1994; de Zambotti et al., 2016). We compared the FBCHR and pPSG measurements of HR on a minute‐by‐minute basis (Figure 1).

We computed the mean and the standard deviation (*SD*) of the single‐epoch differences between the pulse detected by the FBCHR and the pPSG. The same calculation was repeated for each vigilance state (i.e. sleep stages and wake). Pearson’s correlation coefficient was computed for each participant between paired data that were not stratified for vigilance state, and then for all sleep stages and waking (Giavarina, 2015). Correlation coefficients were computed separately in the two groups identified by PSG’s HR ≥100 bpm and PSG’s HR <100 bpm.

To formally test whether the correlation between paired data varied for different HR zones, as graphically suggested by the Bland–Altman plots, correlations between the pPSG and Fitbit‐detected HR were re‐computed for bins of 10‐bpm intervals.

Along with Pearson’s correlation coefficients, we calculated both the Lin’s concordance correlation coefficients (CCC) and the mean absolute percentage errors (MAPE).

A Bland–Altman analysis (Aadland & Ylvisaker, 2015; Nelson & Allen, 2019) was performed in order to visually assess the distribution of the difference between HR measured by the FBCHR and pPSG. Both Bland–Altman plots and limits of agreement (LoA) were computed for each sleep stage and in wakefulness.

To understand how movement, detected as actiometric measures, might affect the HR detection of the FBCHR, we estimated a quantile regression model with response ΔHR = (HR_FITBIT_ − HR_PSG_) and the following predictors: the PSG itself, and a natural cubic spline that describes the effect of the actiometric measures.

## 3 RESULTS

### 3.1 Correlation Analysis between pPSG and FBCHR HR detection

**FIGURE 2:**
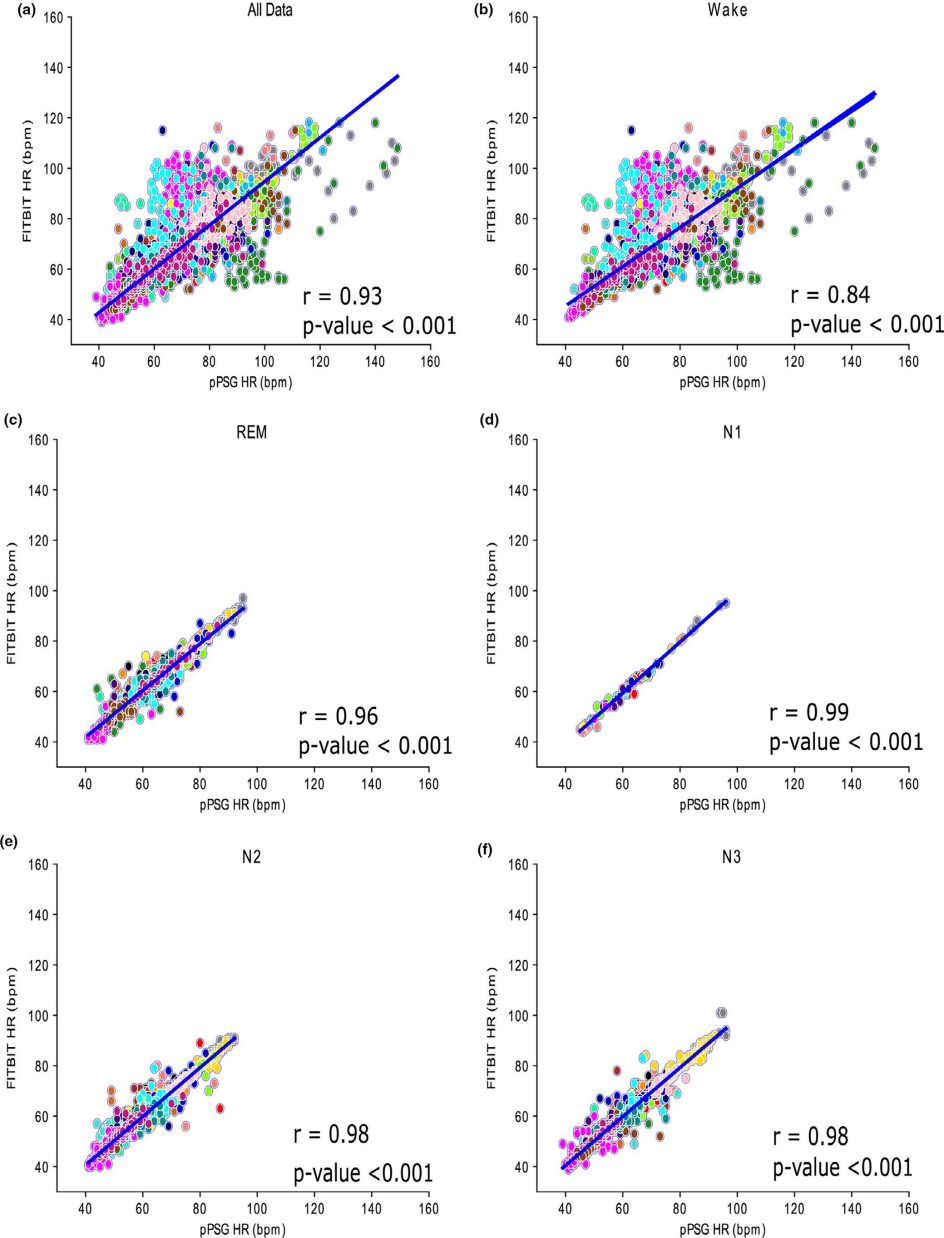
Scatterplots and regression lines for aggregated vigilance states (Panel a), wakefulness (Panel b), rapid eye movement (REM, Panel c), N1 (Panel d), N2 (Panel e) and N3 (Panel f). The corresponding Pearson’s correlation coefficients and *p* values are superimposed to each plot. Each colour displays data obtained from a single subject. Time resolution: 1 min

Pearson’s correlation coefficient (*r*) was computed on aggregated paired HR data (FBCHR and pPSG), showing an overall correlation of 0.93 (number of samples [*n*] = 13,058; Figure 2a). The correlation was lower in wakefulness (*r* = 0.84, *n* = 5,028, Figure 2b), and greater for each and every sleep phase (REM, *r* = 0.97, *n* = 1,656; N1, *r* = 0.99, *n* = 77; N2, *r* = 0.98, *n* = 3,282; N3, *r* = 0.98, *n* = 3,015, Figure 2c–f). In Table 1 the correlation coefficients are reported for each monitored subject, while in Table S1 are shown the CCCs and MAPEs.

**TABLE 1:** Participants’ Pearson’s correlation coefficients (*r*) calculated for aggregated activity states (number of samples [*n*] = 13,058) and for each activity state (Wake, *n* = 5,028; REM, *n* = 1656; N1, *n* = 77; N2, *n* = 3,282; N3, *n* = 3,015). The number of outliers is shown in brackets

ID	Age, years	Samples, *n*	Whole recording	Wake (outliers)	REM (outliers)	N1 (outliers)	N2 (outliers)	N3 (outliers)
1	21	452	0.89	0.92 (12)	0.69 (3)	0.91 (0)	0.87 (0)	0.84 (0)
2	22	586	0.69	0.65 (66)	0.96 (0)	0.98 (0)	0.90 (0)	0.88 (0)
3	24	540	0.84	0.77 (42)	0.83 (0)	1 (0)	0.50 (3)	0.50 (6)
4	21	597	0.93	0.91 (27)	0.91 (3)	1 (0)	0.82 (3)	0.95 (0)
5	20	663	0.83	0.77 (30)	0.86 (0)	0.83 (0)	0.79 (6)	0.86 (0)
6	20	517	0.81	0.78 (33)	0.72 (0)	0.59 (0)	0.73 (3)	0.83 (3)
7	23	565	0.89	0.84 (66)	0.86 (3)	0.97 (0)	0.68 (9)	0.69 (3)
8	22	605	0.92	0.86 (87)	0.92 (0)	1 (0)	0.95 (0)	0.95 (0)
9	20	316	0.92	0.93 (0)	0.97 (0)		0.93 (0)	0.88 (3)
10	30	348	0.88	0.80 (21)	0.86 (0)		0.89 (0)	0.93 (0)
11	22	692	0.94	0.91 (90)	0.95 (0)	0.65 (0)	0.94 (3)	0.96 (0)
12	30	528	0.91	0.89 (3)	0.90 (3)		0.77 (0)	0.86 (0)
13	24	441	0.77	0.62 (183)	0.68 (12)	1 (0)	0.93 (0)	0.79 (3)
14	21	547	0.82	0.55 (54)	0.85 (3)	0.87 (0)	0.85 (0)	0.86 (3)
15	20	649	0.89	0.86 (21)	0.89 (0)	0.98 (0)	0.81 (6)	0.80 (3)
16	21	580	0.91	0.89 (33)	0.92 (0)		0.96 (0)	0.97 (0)
17	22	583	0.65	0.57 (36)	0.81 (0)	0.97 (0)	0.74 (3)	0.78 (3)
18	21	564	0.81	0.74 (81)	0.91 (3)	1 (0)	0.93 (0)	0.83 (3)
19	22	376	0.93	0.88 (51)	0.84 (0)	0.82 (0)	0.88 (0)	0.88 (0)
20	20	473	0.91	0.82 (168)	0.75 (3)	0.98 (0)	0.83 (0)	0.76 (0)
21	21	605	0.79	0.71 (33)	0.94 (0)		0.93 (0)	0.88 (0)
22	20	250	0.95	0.95 (27)	0.36 (3)		0.95 (0)	0.55 (6)
23	29	694	0.48	0.47 (105)	0.73 (0)		0.66 (6)	0.44 (3)
24	24	370	0.84	0.87 (21)	0.73 (0)		0.76 (9)	0.83 (0)
25	19	517	0.91	0.78 (12)	0.99 (0)		0.94 (3)	0.93 (0)
Mean [*SD*] (outliers)	22.36 [3.00]	522.32 [115.71]	0.84 [0.11]	0.78 [0.13] (1,302)	0.83 [0.130] (36)	0.91 [0.14] (0)	0.83 [0.11] (54)	0.82 [0.14] (36)

ID, participant identification number; REM, rapid eye movement.

**FIGURE 3:**
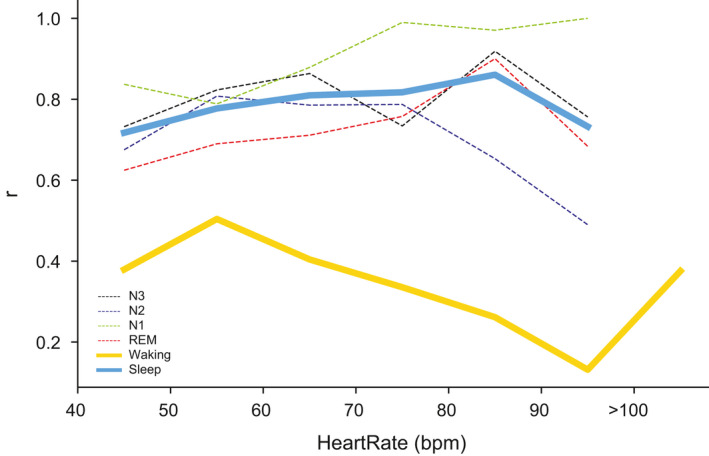
Pearson’s correlation coefficients (*r*) plotted against heart rate ranges. Correlation coefficients between Fitbit ChargeHR™ (FBCHR) and portable polysomnographic (pPSG) paired data are higher in aggregated (large blue solid line) as well as in each sleep stage, plotted separately (rapid eye movement [REM], thin red dashed line; N1, thin green dashed line; N2, thin blue dashed line; N3, thin dashed black line) than waking (large solid yellow line), across all heart rate frequency bins

For the same HR intervals, the correlation was consistently higher during sleep than waking (Figure 3). Moreover, epochs with HRs >100 bpm showed a significantly worse correlation (*r* = 0.35, *n* = 116) than those with HRs <100 bpm (*r* = 0.84, *n* = 4,912).

### 3.2 Bland–Altman analysis

**FIGURE 4:**
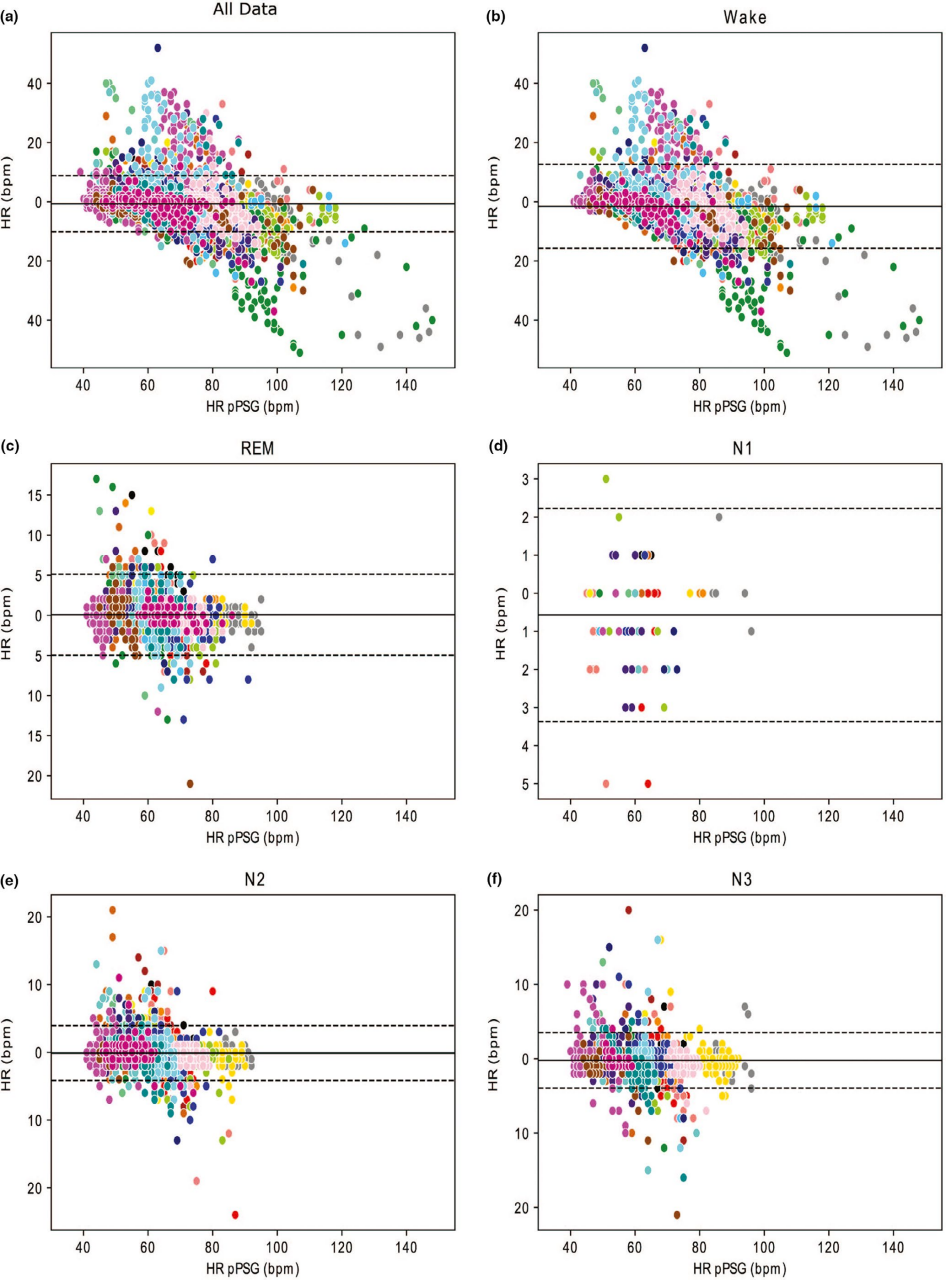
Bland–Altman plots for aggregated vigilance states (Panel a), Wakefulness (Panel b), rapid eye movement (REM, Panel c), N1 (Panel d), N2 (Panel e) and N3 (Panel f). Mean bias (thick solid line) and upper and lower limits of agreement (mean ± 1.96 *SD*, thin dotted lines) are shown. Each colour displays data obtained from a single subject. Time resolution: 1 min. The *y*‐axis represents the difference between the heart rate (HR) computed based on Fitbit ChargeHR™ (FBCHR) and the HR computed based on portable polysomnographic (pPSG) (ΔHR = HR_FITBIT_ − HR_PSG_ bpm), while the *x*‐axis is the HR detected by the pPSG

Overall, regardless of vigilance state, the mean difference of HR measurements between the FBCHR and pPSG was ΔHR = (HR_FITBIT_ − HR_PSG_) = −0.66 *SEM*: 0.04 bpm (Figure 4a). Such bias was larger for wake epochs (ΔHR = −1.51 *SEM*: 0.10 bpm, Figure 4b) compared to each sleep stage (REM, ΔHR = 0.03 *SEM*: 0.06 bpm; N1, ΔHR = −0.29 *SEM*: 0.16 bpm; N2, ΔHR = −0.12 *SEM*: 0.04 bpm; N3, ΔHR = −0.21 *SEM*: 0.04 bpm, Figure 4c–f). As it can be observed in Figure 4, the HR estimation by the FBCHR from 60 to 80 bpm, while it tends to overestimate the HR for lower and upper frequencies, respectively. Furthermore, in Bland–Altman plots (Figure 4) those samples lying at >100 bpm have a more diffuse distribution (i.e. a wider dispersion).

### 3.3 Quantile regression model

**FIGURE 5:**
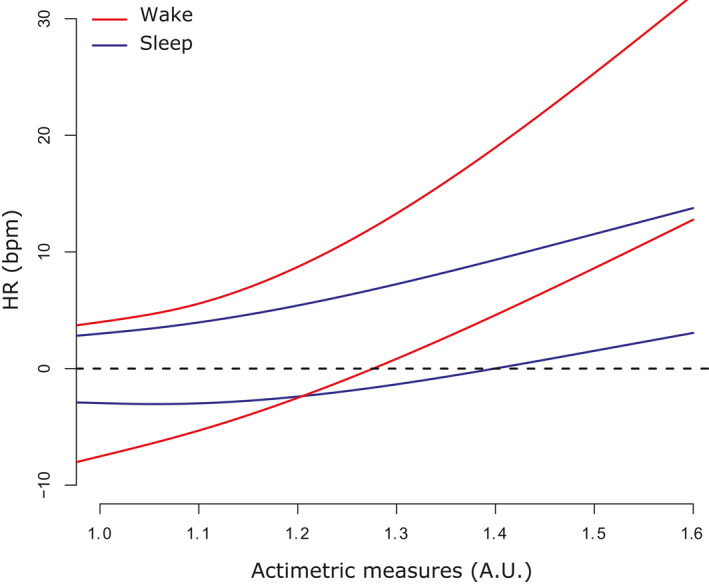
Quantile regression model estimated with response Fitbit ChargeHR™ (FBCHR)‐portable polysomnographic (pPSG) and the following predictors: the pPSG itself, and a natural cubic spline that describes the effect of the actiometry. Here are shown the predicted quantiles of order 0.05 and 0.95, for pPSG = 60 bpm

In Figure 5, we show the predicted quantiles of order 0.05 and 0.95, for pPSG = 60 bpm. The bias of the FBCHR is generally positive and is closer to zero at low actiometric measures. While the bias is an increasing function of the actiometric measures, the rate of increase is much higher during wake than during sleep.

## 4 DISCUSSION

In the present study the accuracy of pulse estimation by the FBCHR is evaluated in comparison to the HR estimated by the pPSG under free‐living conditions (Aadland & Ylvisaker, 2015; Nelson & Allen, 2019). Our present results show that the FBCHR tends to underestimate the HR detected by the pPSG ECG recording (overall Bland–Altman bias = −0.66 *SEM*: 0.04 bpm). This evidence is supported by other studies suggesting that the FBCHR’s PurePulse is prone to underestimate the HR compared to the HR detected on ECG traces recorded by PSG devices (de Zambotti et al., 2016), chest‐worn three‐lead ECG (Haghayegh et al., 2019) and ambulatory ECG (Nelson & Allen, 2019). The bias is larger during wake than during sleep phases (Waking Bland–Altman bias = −1.514 *SEM*: 0.10 bpm). Consistently, also the correlation coefficient between the pPSG and FBCHR measurements is at its peak during wakefulness compared to sleep. The observation that the FBCHR is less reliable when the wearer is awake is consistent with other studies (Jo et al., 2016; Reddy et al., 2018). The higher wrist movement rates during waking and the related lower stability of the sensor contact with the skin during wakefulness can account for the lower agreement between the FBCHR and pPSG during wake, as suggested by the quantile regression model. The impact of movement on wrist photoplethysmographic HR detection has been already proposed by Benedetto et al., (2018) and by Bent et al., (2020). Instead, we exclude that the difference in accuracy between sleep and waking might depend on the mean difference in frequency rates between vigilance states. In fact, after controlling for the HR ranges, the FBCHR remains more accurate during sleep as compared to waking. Moreover, the correlation between HR measured by the FBCHR and pPSG highlights the low reliability of FBCHR in estimating HR when the pulse is >100 bpm. The accuracy of the FBCHR during moderate to high physical activity states is controversial in the literature: some studies (Bai et al., 2018; Reddy et al., 2018) indicate an accurate HR estimation by the FBCHR PurePulse technology. Other studies (Bent et al., 2020; Jo et al., 2016) also support our conclusions: the FBCHR could not be considered as a reliable HR estimator of the HR during fitness activities. In the same paper, along with Sjoding et al., (2020), the authors show how the skin tone does impair the accuracy of the detection. In our study, given the demographics of the subjects enrolled, we could not verify this claim.

Consistently with the correlation coefficients results, the LoA of Bland–Altman analysis (Figure 4) are wider for waking samples compared to sleep LoA. This result suggests that the HR measurements of the FBCHR tends to be more accurate (i.e. nearer to the HR measured by the pPSG) in sleep epochs compared to wakefulness.

Both the low accuracy in wakefulness and the reliability of HR estimation in sleep have multiple implications. The FBCHR can provide clinicians a feasible tool for accurately and continuously monitor HR during sleep. Beyond the clinical applications, it can be exploited in basic sleep research protocol, especially in those studies investigating HR variability and the autonomic nervous system. Also, the FBCHR could improve the analysis of sleep parameters such as wake after sleep onset or sleep efficiency. The FBCHR can possibly be used in HR monitoring during resting wakefulness, when the low rate of rest movements affects the contact between the PurePulse diode and the skin less. Because PurePulse LED technology is shared with other Fitbit Inc. Smartbands, our results are generalisable to other devices (Haghayegh et al., 2019). A limitation of our present study is that it was conducted on a relatively small sample, entirely composed of Caucasian, healthy young adults, the majority of whom were females. Furthermore, we computed the correlation coefficients for HR >100 bpm on 116 paired samples. The small sample size could further affect our results and our conclusion on the FBCHR reliability in this frequency range.

In conclusion, in the present study we addressed the quality of HR detection of a Fitbit Inc. wrist‐worn Smartbands device (Fitbit ChargeHR^™^) across all vigilance states (i.e. all sleep stages and waking). We conducted this quality assessment through different statistical methodologies, such as correlation coefficient computation and Bland–Altman analysis with a 1‐min temporal resolution. Our present results indicate that the accuracy of the tested Smartband is substantially higher during sleep than in waking, across all HR zones. A plausible explanation is provided by the motion‐related artefacts occurring during waking.

## CONFLICTS OF INTEREST

UO and UF are co‐founders of sleepActa S.r.l., a spin‐off company of the University of Pisa operating in the field of sleep medicine. All other authors declare no potential conflict of interest.

## AUTHOR CONTRIBUTIONS

**DB:** Data collection, investigation, formal analysis, methodology, software, visualisation, writing – original draft, writing – review and editing. **UO:** Methodology, software, resources, validation, supervising, visualisation, writing – review and editing. **PF:** Investigation, methodology, software, formal analysis, visualisation, writing – review and editing. **AB:** Visualisation, validation, writing – review and editing. **SB:** Visualisation, validation, writing – review and editing. **Pd’A:** Validation, writing – review and editing. **MM:** Resources, validation, writing – review and editing. **EB:** Resources, validation, writing – review and editing. **UF:** Conceptualisation, project administration, funding acquisition, resources, supervision, validation, visualisation, writing – review and editing.

## Supporting information

Table S1

## Data Availability

Due to the nature of this research and according to the informed consent signed by the participants, supporting data are available only in aggregated form, according to the protocol approved by the Bioethical Committee of the University of Pisa (Review No: 02/2020 Prot. 0036352/2020).
