# Supplementary material for: Heart rate detection by Fitbit ChargeHR™: A validation study versus portable polysomnography
Source: J Sleep Res. 2021 Apr 10;30(6):e13346. doi: 10.1111/jsr.13346 (PMC9286609; doi:10.1111/jsr.13346)
Supplement: Supplementary file 1 — Table S1 [file JSR-30-0-s001.docx]

| Data | CCC estimate  (95 -CI) | MAPE |
| --- | --- | --- |
| Overall | 0.90 (0.89-0.91) | 3.38 |
| Wakefulness | 0.83 (0.83-0.84) | 5.47 |
| REM | 0.97 (0.96-0.97) | 2.76 |
| N1 | 0.99 (0.99-0.99) | 1.83 |
| N2 | 0.98 (0.98-0.98) | 2.01 |
| N3 | 0.98 (0.98-0.99) | 1.74 |

TABLE S1. The Lin’s Concordance Correlation Coefficient (CCC) and the Mean Absolute Percentage Error (MAPE) are calculated for aggregated activity states and for each activity state.
